# Maternal Microbiome in Fetal Programming: A One Health Perspective on Translational Implications for Early-Life Health

**DOI:** 10.3390/microorganisms14061214

**Published:** 2026-05-27

**Authors:** Mariarosaria Matera, Valentina Biagioli, Ilaria Cavecchia, Maria Teresa Illiceto, Laura Pennazzi, Matilde Morandin, Maria Beatrice Lenzi, Maria Elisabetta Baldassarre, Maurizio Mennini

**Affiliations:** 1 Department of Pediatric Emergencies, Misericordia Hospital, 58100 Grosseto, Italy; 2 Italy Microbiota International Clinical Society (MICS), 10153 Turin, Italy; 3 Department of Neuroscience, Rehabilitation, Ophthalmology, Genetics, Maternal and Child Health, University of Genoa, 16126 Genoa, Italy; 4 Microbiomic Department, Koelliker Hospital, 10134 Turin, Italy; 5 Unit of Pediatric Gastroenterology and Digestive Endoscopy, Department of Pediatrics, Santo Spirito Hospital, 65125 Pescara, Italy; 6 DeaLuce Obstetric/Nutritional Practice, 00168 Rome, Italy; 7 Department of Neonatology and NICU, Villa Sofia-Cervello Hospital, 90146 Palermo, Italy; 8 Interdisciplinary Department of Medicine, Neonatology and Neonatal Intensive Care Unit, Aldo Moro University, 70121 Bari, Italy; 9 Department of Neurosciences, Mental Health and Sensory Organs (NESMOS), Sapienza University of Rome, 00185 Rome, Italy

**Keywords:** fetal programming, maternal microbiome, environmental factors, mother health

## Abstract

Pregnancy represents a critical eco-biological window during which maternal physiology integrates environmental exposures, lifestyle factors, and interconnected microbial ecosystems to shape fetal development and long-term health. From a One Health perspective, defined here as the interconnection between maternal health, environmental determinants, and microbial ecosystems across generations, the maternal microbiome functions as a dynamic interface linking the external environment to the intrauterine milieu, translating ecological signals into immunological, metabolic, and neuroendocrine pathways that influence placental function and developmental programming. Across gut, vaginal, oral, and mammary niches, maternal microbial communities operate as an integrated network regulating systemic inflammation, metabolic homeostasis, and the production of bioactive metabolites, including short-chain fatty acids, bile acids, and tryptophan derivatives. This review proposes an integrated systems framework in which pregnancy is viewed as a transient ecological system shaped by ten interconnected maternal determinants, encompassing microbial niches, nutrition, lifestyle factors, medical interventions, mode of delivery, and postnatal microbial transmission, that converge on shared microbiome-mediated signaling pathways affecting fetal and neonatal immune, metabolic, and neurodevelopmental trajectories. Broader macro-environmental drivers, including biodiversity loss, urbanization, pollution, and industrialized lifestyles, are considered as upstream modulators of maternal microbial ecology within a One Health context. A systems model is presented to illustrate how environmental inputs are biologically transduced through maternal microbial networks to influence placental function, fetal development, and early-life health trajectories. Framing pregnancy as an integrated eco-biological continuum highlights the maternal microbiome as a central hub of intergenerational health and may support microbiome-informed preventive strategies and public health approaches aimed at reducing the burden of non-communicable diseases (NCDs) of early-life origin.

## 1. Introduction

Pregnancy represents a critical and time-limited biological window during which maternal physiology undergoes profound immunological, metabolic, endocrine, and vascular adaptations to sustain fetal growth and development. Gestation is increasingly conceptualized as a dynamic eco-biological system in which maternal tissues, environmental exposures, and microbial ecosystems interact continuously rather than as independent domains [1,2]. Within this framework, the maternal organism functions as an interface between the external environment and the developing fetus, integrating ecological signals, including the maternal microbiota, into biological processes that shape intrauterine conditions [3].

The One Health paradigm, originally developed to recognize the interdependence of human, animal, and environmental health, has progressively expanded toward systems-based models of health across ecological contexts. In the present review, we apply One Health as an integrative framework through which pregnancy can be understood as a multilevel system influenced by broader ecological determinants and a pivotal stage for shaping fetal and lifelong wellbeing [4]. Within this perspective, maternal biology, environmental exposures, and microbial ecosystems are considered interconnected drivers of offspring developmental trajectories. Dietary patterns, physical activity, psychosocial stress, medical interventions, and environmental exposures converge on shared maternal physiological pathways. Among these integrative systems, the maternal microbiome has emerged as a central mediator [3]. Microbial communities across the gut, vaginal, oral, and mammary niches constitute a metabolically active network that modulates systemic inflammation, immune function, endocrine signaling, and metabolic homeostasis [5]. Perturbations in these communities, including dysbiosis or maternal immune activation (MIA), have been linked to altered fetal neurodevelopment and adverse postnatal outcomes [6,7,8,9]. Clinically overt inflammatory conditions such as maternal sepsis, chorioamnionitis, and amniotic fluid infection further illustrate how disrupted maternal host–microbial interactions may adversely affect fetal development, neonatal immune responses, and adaptation to extrauterine life [10,11,12].

Recent evidence suggests a functional reproductive-microbiota-fetal (RMF) axis, whereby intestinal microbial communities influence reproductive physiology, placental function, and fetal development via immune, metabolic, and endocrine pathways [3]. Microbial metabolism generates bioactive compounds, including short-chain fatty acids (SCFAs), bile acids (BA), and tryptophan derivatives (TD), which regulate host immunity, epithelial barrier integrity, and neuroendocrine signaling [13,14]. These signals may reach the placenta, now recognized as a dynamic immunometabolic interface capable of sensing and responding to maternal environmental cues [15]. This cascade links environmental exposures to fetal development through maternal physiology and microbial activity. Conceptually, pregnancy may be interpreted as a multilevel eco-biological system in which macro-environmental determinants, maternal physiology, and microbial ecosystems continuously interact across gestation [16]. Within this systems-based framework, upstream ecological exposures—including diet, lifestyle, psychosocial stress, environmental pollutants, medical interventions, and patterns of microbial contact—shape maternal microbial ecology across gut, vaginal, oral, and mammary niches [1,2]. In turn, the maternal microbiome functions as a dynamic signaling network generating metabolites, immune mediators, and endocrine-modulating compounds capable of influencing placental function and fetal developmental pathways [1,2,13,14,15,16,17,18,19,20]. The placenta operates as an immunometabolic interface integrating these maternal signals and translating them into coordinated fetal responses affecting immune maturation, metabolic homeostasis, and neurodevelopment [21,22]. This integrated model supports the interpretation of fetal programming not as an isolated intrauterine phenomenon, but as the biological consequence of interconnected ecological and maternal-host interactions operating across critical developmental windows [3].

Fetal development exhibits marked plasticity. According to the Developmental Origins of Health and Disease framework, postnatal-life conditions can induce long-lasting structural, metabolic, and epigenetic adaptations influencing disease susceptibility across the lifespan [23,24]. Maternal immune activation represents a first “hit” priming fetal immune and neurodevelopmental systems, whereas subsequent adverse postnatal exposures may act as additional “hits”, driving maladaptive epigenetic and immune responses that increase long-term disease risk [7]. While nutritional and endocrine determinants have been extensively studied, microbiome-mediated pathways remain insufficiently integrated into research models and clinical practice, despite evidence linking early microbial exposures to allergic diseases, asthma, obesity, metabolic disorders, and neurodevelopmental conditions [25,26]. Integrated models simultaneously considering macro-environmental drivers, maternal microbial ecology, placental biology, and fetal programming remain limited, and translation into routine obstetric care is incomplete [26,27]. Microbiota-targeted strategies aimed at modulating maternal-fetal signaling pathways are under investigation but are not yet part of standard clinical practice [28]. Given the rapid expansion of literature across microbiome science, obstetrics, developmental biology, and environmental health, current evidence remains fragmented across disciplines. An extensive integrative review is therefore warranted to synthesize these converging data within a unified maternal-fetal systems framework.

This review adopts a One Health-informed systems perspective to synthesize current evidence on microbiome-mediated maternal-fetal programming. Specifically, the review examines how maternal microbial ecosystems integrate environmental, lifestyle, clinical, and biological determinants into signaling pathways capable of influencing placental function and early-life developmental trajectories. By combining microbial ecology, fetal programming, placental biology, and macro-environmental determinants within a unified conceptual framework, this review aims to provide a more integrated interpretation of maternal-fetal health across critical developmental windows and to highlight potential translational implications for preventive maternal-child healthcare.

## 2. Literature Search and Study Selection

### 2.1. Study Design

This work is a narrative review supported by a structured and targeted but non-systematic literature search. Its objective was to develop a conceptual, microbiome-centered framework of maternal-fetal programming from a One Health perspective. The review integrates current evidence on the maternal microbiome as a biological interface linking environmental exposures, maternal physiology, and fetal developmental trajectories, with emphasis on immune, metabolic, and neurodevelopmental outcomes. As this is a conceptual synthesis rather than a systematic or exhaustive evidence appraisal, no formal systematic review protocol was undertaken.

### 2.2. Literature Search

A structured literature search was conducted to identify relevant studies published between January 2015 and January 2026 to capture the most recent evidence on the maternal microbiome and its role in fetal programming. Earlier landmark studies were also included when necessary to provide biological background, mechanistic insights, or conceptual support. These were identified through backward citation tracking and manual searches based on key authors and seminal publications.

The search focused on peer-reviewed articles investigating maternal microbiota across different body sites (gut, vaginal, oral, and mammary), microbiome-derived metabolites, maternal-fetal signaling pathways, and environmental and lifestyle determinants influencing microbial ecology during pregnancy. The search strategy was designed to ensure conceptual coverage rather than exhaustive retrieval of all available studies.

### 2.3. Study Selection

A targeted literature search was conducted in major biomedical databases to identify relevant studies on the maternal microbiome during pregnancy and its role in maternal–fetal programming within a One Health framework. Titles and abstracts were screened by the authors for relevance, and full-text articles were selected based on their contribution to understanding the maternal microbiome as a biological interface linking environmental exposures, maternal physiology, and fetal outcomes, particularly in the domains of immune, metabolic, and neurodevelopmental programming. Due to the narrative and conceptual nature of this review, study selection was not conducted according to a formal systematic review protocol, and no quantitative synthesis was performed.

A total of 200 records were initially identified through database searches conducted in PubMed, Scopus, and Web of Science. The search strategy combined keywords related to “maternal microbiome”, “pregnancy”, “fetal programming”, “immune development”, “metabolic outcomes”, and “neurodevelopment”. After removal of duplicates and screening based on titles and abstracts, a subset of studies was assessed in full text for eligibility.

Studies were included if they addressed at least one of the following domains:

(i) maternal microbiome composition and function;
(ii) microbial metabolites and signaling pathways relevant to fetal development;
(iii) maternal-fetal immune, metabolic, or neuroendocrine interactions;
(iv) environmental, lifestyle, or clinical determinants influencing maternal microbial ecology;
(v) early-life microbial transmission and postnatal continuity.

Both experimental and observational studies, as well as high-quality reviews and meta-analyses, were considered.

### 2.4. Data Extraction

For each eligible study, key information was extracted, including study design, population characteristics (maternal and/or infant), type of microbial ecosystem investigated, relevant exposures (diet, stress, physical activity, antibiotics, environmental factors), and main outcomes related to fetal programming or early-life health.

Additional data regarding microbial metabolites (e.g., short-chain fatty acids, bile acids, tryptophan derivatives), immune pathways, and placental mechanisms were also collected. Data extraction was conducted through iterative reading and synthesis of the literature by the authors. Data were interpreted within a systems-based framework to identify recurring biological pathways linking maternal microbiome dynamics to fetal and neonatal outcomes.

### 2.5. Development of the Conceptual Framework

The proposed framework of microbiome-mediated maternal-fetal programming was developed through an iterative conceptual synthesis of the selected literature. Key determinants influencing maternal microbial ecology and downstream fetal outcomes were identified and organized into interconnected ecological domains.

These domains include maternal microbial niches, lifestyle and clinical factors, environmental exposures, and postnatal microbial continuity, all integrated within a One Health perspective. The framework was progressively refined through critical discussion among the authors to reflect the multidimensional interactions linking environment, microbiome, placenta, and fetal development.

The resulting model conceptualizes pregnancy as a dynamic eco-biological system in which the maternal microbiome acts as a central hub translating environmental inputs into biological signals that shape early-life health trajectories.

## 3. Biological Foundations of Maternal-Fetal-Infant Microbial Interactions

### 3.1. Dynamic Changes in the Maternal Microbiome Across Gestation and Transmission Pathways

Pregnancy is associated with progressive remodeling of maternal microbial ecosystems rather than a static microbiome state [1,2]. Across gestation, endocrine, immunological, metabolic, and behavioral changes reshape microbial communities in a niche-specific manner [29,30]. These adaptations may alter the production of microbial metabolites, inflammatory mediators, and endocrine signals that can be conveyed through maternal circulation, placental interfaces, birth processes, and postnatal breastfeeding pathways, thereby influencing fetal development and neonatal adaptation [21,22,23,24,25,26,27,28,29,30,31,32]. These processes support the concept of microbiome-mediated signal translation across the maternal-fetal interface.

Maternal microbial ecosystems constitute the primary source of postnatal life microbial exposure. Neonatal colonization is increasingly understood as a structured ecological succession shaped by maternal reservoirs and host-microbe coevolution rather than a stochastic process [33]. Strain-resolved metagenomics demonstrates extensive vertical transmission from multiple maternal body sites, identifying the mother as the dominant microbial donor in early life [17,18].

During vaginal delivery, neonates initially acquire microbes from vaginal communities enriched in *Lactobacillus*, contributing to early mucosal and cutaneous colonization [34]. These delivery-associated transmission patterns are further discussed from an ecological and clinical perspective in Section 4.6. These taxa suddenly decline within weeks and are replaced by obligate anaerobes characteristic of the intestinal ecosystem [18]. Cesarean birth reduces transmission of maternal gut anaerobes, delays microbiota maturation, and increases colonization by environmental microbes, changes associated with later immune and metabolic outcomes [35]. However, transmission is not simply diminished but redistributed across maternal niches, with gut, skin, and breast milk contributing in a coordinated, delivery-dependent manner [17].

The maternal intestinal microbiota represents the principal reservoir guiding neonatal gut assembly. Importantly, this microbial reservoir includes beneficial, commensal, and potentially pathogenic microorganisms, reflecting a spectrum of effects on host physiology. Persistent mother-infant strain sharing indicates sustained transmission beyond delivery [17,18]. Breastfeeding reinforces this continuity: human milk provides a complex and dynamic mixture of microbial and bioactive components, together with human milk oligosaccharides (HMOs) that selectively promote the expansion of beneficial taxa, particularly *Bifidobacterium*, in the infant gut [36,37]. In addition to microbes and HMOs, human milk also delivers immunologically active components, including immunoglobulins, which contribute to neonatal immune development and reflect maternal environmental exposures. An entero-mammary pathway further supports the transfer of maternal gut microbes via immune cell trafficking [36]. In cesarean-born infants, partial restoration of maternal microbial transfer through breastfeeding or experimental maternal fecal microbiota transplantation may accelerate microbiota maturation toward vaginally delivered profiles, although these approaches remain investigational [17,18,19,20,21,22,23,24,25,26,27,28,29,30,31,32,33,34,35,36,37,38]. In utero microbial colonization remains controversial. Although bacterial DNA has been detected in placental or fetal tissues [30,31,32,33,34,35,36,37,38,39], rigorous analyses attribute many findings to contamination, and no consistent placental microbiome has been confirmed [40,41]. Prenatal influences are therefore more plausibly mediated by immune and metabolic signals crossing the placenta, rather than by direct microbial colonization [21,22]. These include the transfer of immunoglobulins and other immune mediators, whose composition may vary in response to maternal environmental exposures. Furthermore, maternal inflammatory conditions, such as infections or sepsis, may influence fetal development through circulating mediators, including lipopolysaccharide (LPS), contributing to fetal immune programming. Collectively, these pathways highlight how maternal microbial ecosystems influence early-life biology not only through vertical transmission but also through systemic signaling mechanisms operating across the maternal-fetal interface.

### 3.2. Microbial Metabolites as Developmental Signals

Maternal microbiota influence fetal development through circulating microbial metabolites that function as endocrine-like signals, independent of direct fetal colonization. Experimental evidence demonstrates that pregnancy-associated microbial products can cross the placental barrier or modulate maternal physiology in ways that shape fetal immune and metabolic programming [22,23,24,25,26,27,28,29,30,31,32,33,34,35,36,37,38,39,40,41,42,43]. This paradigm reframes the maternal microbiome as a systemic signaling hub rather than merely a source of neonatal inoculum. SCFAs, primarily acetate, propionate, and butyrate, are the most extensively studied mediators. Produced by microbial fermentation of dietary fibers, SCFAs have been shown to modulate fetal immune maturation, promote regulatory T-cell development, and influence energy metabolism through G-protein-coupled receptors and histone deacetylase inhibition [44]. Experimental studies in murine models demonstrate causal effects on offspring metabolic phenotype [45], while human cohort studies have associated higher maternal fiber intake and SCFA-related microbial profiles with reduced risk of allergic disease and asthma in children [46].

Microbial metabolism of tryptophan generates indole derivatives that activate the aryl hydrocarbon receptor (AhR), a key regulator of barrier integrity and immune differentiation. These compounds contribute to mucosal immune development and tolerance pathways essential for early-life host-microbe symbiosis [42,43,44,45,46,47]. Microbiota also transform primary bile acids into secondary bile acids, which act as signaling molecules via nuclear and membrane receptors such as FXR and TGR5. These pathways influence lipid and glucose metabolism and may modulate fetal hepatic and intestinal development [1,2,13,14,15,16,17,18,19,20,21,22,23,24,25,26,27,28,29,30,31,32,33,34,35,36,37,38,39,40,41,42,43,44,45,46,47,48].

Finally, low-level microbial components, including LPS and other microbe-associated molecular patterns (MAMP), can provide physiological immune priming rather than pathological inflammation. Controlled exposure to such signals is thought to calibrate innate immune responsiveness before birth [19]. Together, these findings support a model in which maternal microbial ecosystems shape fetal development through a diverse repertoire of bioactive metabolites, establishing early immune and metabolic trajectories. Emerging evidence suggests that these prenatally established microbial and metabolic signals influence postnatal microbial and metabolite profiles, which are associated with long-term outcomes, including infant immune function and neurodevelopment extending into childhood and adolescence [49,50,51].

### 3.3. Placenta as an Immunometabolic Interface

The traditional concept of a sterile intrauterine environment has been progressively revised, supporting a functional interpretation of sterility as the absence of stable colonization rather than of biological signals [21,22,23,24,25,26,27,28,29,30,31,32,33,34,35,36,37,38,39,40,41,42,43,44,45,46,47,48,49,50,51,52,53]. While in utero colonization remains debated, growing evidence highlights a role for microbial-derived components in shaping early immune development [54]. The placenta represents the central hub of this axis, functioning as a dynamic immunometabolic interface that senses, integrates, and transduces maternal environmental, microbial, and fetal signals to regulate fetal development [55]. Beyond nutrient transport and gas exchange, it orchestrates immune tolerance, inflammatory signaling, and metabolic programming, shaping fetal organogenesis and long-term health trajectories [1,2,15,16,17,18,19,20,21,22,23,24,25,26,27,28,29,30,31,32,33,34,35,36,37,38,39,40,41,42,43,44,45,46,47,48,49,50,51,52,53,54,55,56]. Maternal-fetal immune tolerance is mediated by specialized placental cell populations, including extravillous trophoblasts and decidual immune cells, which express pattern recognition receptors (PRRs) and secrete immunomodulatory cytokines. These mechanisms prevent maternal immune rejection while allowing controlled responsiveness to microbial metabolites and inflammatory cues [57,58]. Microbial metabolites present during gestation, including SCFAs and tryptophan derivatives, originating in part from the maternal microbiota, may reach the placental environment and contribute to immune calibration at the maternal-fetal interface [42,43,44,45,46,47]. Placental immune cells, including decidual macrophages and uterine natural killer (NK) cells, integrate maternal signals to regulate immune tolerance, trophoblast invasion, and vascular remodeling at the maternal-fetal interface, thereby contributing to an intrauterine immune milieu that may help shape fetal immune development [59,60].

Inflammatory pathways within the placenta respond to microbial-associated molecular patterns (MAMPs) and maternal cytokines. Low-level exposure to microbial products, including SCFAs and LPS-derived signals, can activate NF-κB and MAPK cascades without triggering pathological inflammation, thereby potentially calibrating fetal innate immune readiness [19,20,21,22]. Dysregulation, however, may lead to placental dysfunction, altered nutrient transport, and adverse developmental outcomes [56]. Metabolic sensing is another critical placental function. Placental cells detect and respond to maternal-derived metabolites, including SCFAs, maternal circulating bile acids, and amino acid derivatives, adjusting nutrient fluxes, mitochondrial activity, and energy metabolism to optimize fetal growth [61,62]. While maternal bile acid metabolism is partly shaped by gut microbial activity, direct evidence for placental transfer of microbiota-derived secondary bile acids to the fetus remains limited. These metabolite-mediated signals, established prenatally, are also suggested to influence epigenetic programming in trophoblasts and fetal tissues and may shape postnatal immune and neurodevelopmental trajectories [42,43,44,45,46,47,48,49,50,51].

Collectively, the placenta operates as a central integrator of maternal microbiota-derived metabolites, immune cues, and environmental factors, translating complex maternal signals into coordinated fetal developmental responses. Its immunometabolic interface ensures tolerance, calibrates inflammation, and modulates epigenetic landscapes, thereby establishing foundational trajectories for immune competence, metabolic homeostasis, and neurodevelopment, independent of direct fetal microbial colonization. These processes highlight the placenta as a key biological interface through which maternal microbial ecosystems influence fetal development within a One Health framework.

## 4. Ten Ecological Determinants of Microbiome-Mediated Maternal-Fetal Programming: A One Health Perspective

The ten ecological determinants presented in this section should not be interpreted as independent or isolated factors. Rather, they represent interconnected components of a dynamic and integrated system in which maternal microbiota, host physiology, and environmental exposures continuously interact.

Within a One Health framework, each determinant influences and is influenced by others, forming a multidirectional network of signals that converge at the maternal-fetal interface. For example, diet modulates gut microbial composition and metabolite production, which in turn interacts with immune, endocrine, and neurodevelopmental pathways; similarly, stress, physical activity, and medical exposures (e.g., antibiotics) may reshape microbial ecosystems and alter downstream signaling.

Accordingly, the following subsections are organized for clarity, but should be interpreted as functionally interconnected processes contributing to microbiome-mediated maternal-fetal programming. These determinants exert their effects primarily through modulation of maternal microbial ecosystems and the downstream generation of bioactive signals that are translated across the maternal-fetal interface, as described in Section 3 (Table 1).

### 4.1. Microbial Communication Axis Between Mother and Fetus

Pregnancy represents a critical window of biological communication in which maternal, environmental, and microbial signals converge to shape fetal development [134,135]. In this framework, pregnancy functions as an integrated ecological system linking maternal exposures to developmental trajectories. The maternal-fetal interface functions as a dynamic conduit through which immune mediators, microbial metabolites, and microbiota-derived signals are transferred or detected, thereby contributing to early-life developmental programming [55]. Maternal microbiota composition, influenced by diet, lifestyle, and environmental exposures, modulates the availability of key signaling molecules, including microbiota-derived metabolites (e.g., short-chain fatty acids and tryptophan derivatives) and immune mediators, which can cross or act at the maternal-placental interface, modulating fetal immune and metabolic pathways [136]. While direct in utero microbial colonization of the fetus remains controversial and not conclusively demonstrated, increasing evidence supports a role for microbial-derived components in shaping early immune development by influencing antigen exposure, cytokine signaling, and innate immune priming at the maternal-fetal interface [63]. In addition, maternal microbial exposures influence the transfer of immunoglobulin G, contributing to early immune priming [134,135,136,137].

Microbial activity in the maternal gut can potentially influence progesterone and estrogen production, thereby affecting placental function and fetal growth [135,138]. These signaling processes extend to epigenetic regulation. Microbiota-derived metabolites can influence DNA methylation and histone modification, thereby modulating gene expression without altering DNA sequence. Such mechanisms support fetal developmental plasticity in response to maternal ecological conditions and highlight the interplay between microbiota, nutrition, and placental epigenetic regulation [139]. Understanding pregnancy as a microbial communication axis shifts the focus from a paradigm of protection to one of active preparation. Within this framework, the maternal microbiota emerges as a modifiable biological determinant of early-life programming and long-term health trajectories.

### 4.2. Oral Microbiome and Systemic Inflammation

Within this interconnected framework, the oral microbiome represents an important upstream source of systemic inflammatory signals that may interact with other maternal determinants influencing the maternal-fetal interface. The oral microbiota represents a complex ecosystem in dynamic equilibrium with the host; however, under dysbiotic conditions, it can become a persistent source of systemic inflammation. Periodontal disease is one of the clearest examples of chronic inflammatory exposure: no longer considered a condition confined to the oral cavity, but rather a true driver of the oral-systemic inflammatory burden. In this context, dysbiotic biofilms and chronic gingival inflammation help maintain a state of low-grade systemic inflammation [64]. In recent years, numerous epidemiological studies have strengthened the association between periodontal disease and adverse pregnancy outcomes, including preterm birth, low birth weight, and preeclampsia. These findings suggest an interaction between oral inflammation and the maternal-fetal environment, potentially involving bacterial translocation or modulation of systemic inflammation [64,65,66]. At the same time, the concept of the oral-systemic axis has evolved, with the oral microbiota contributing to a distributed inflammatory network involving multiple body sites. This paradigm moves beyond the traditional focal infection model, instead highlighting the integration between microbiota, inflammation, and systemic health [67]. An emerging area of interest concerns the role of the oral microbiota in vertical transmission. Recent evidence suggests that oral bacteria may contribute to the composition of the breast milk microbiome and influence early oral colonization in infants, with potential implications for immune and metabolic development [20,21,22,23,24,25,26,27,28,29,30,31,32,33,34,35,36].

In light of these findings, preventive strategies play a central role. Proper oral hygiene, a balanced diet, and the targeted use of oral probiotics represent promising tools to modulate the oral microbiota and reduce systemic inflammatory burden. Early intervention in these areas not only helps prevent periodontal disease but also may support maternal systemic homeostasis during pregnancy [140].

Targeted oral probiotics, such as *Streptococcus salivarius* K12 and M18, or *Limosilactobacillus reuteri*, have demonstrated inhibitory effects against major oral pathogens, reductions in volatile sulfur compounds and gingival inflammation, and potential to support oral eubiosis and modulate oral inflammatory burden [68,69,70,71].

### 4.3. Vaginal Microbiome and Reproductive Ecosystem Stability

The vaginal microbiome plays a central role in reproductive ecosystem stability, with Lactobacillus dominance representing a key marker of ecological resilience. A low-diversity, Lactobacillus-rich microbiota is generally considered an optimal configuration in most populations, maintaining an acidic environment that limits pathogen overgrowth and supports reproductive health [141]. Species such as *Lactobacillus crispatus*, *L. gasseri*, and *L. jensenii* contribute to a protective environment by sustaining low vaginal pH and limiting the overgrowth of opportunistic pathogens [72].

Disruption of this equilibrium, characterized by reduced Lactobacillus abundance and increased microbial diversity, is consistently associated with adverse pregnancy outcomes, particularly preterm birth. Recent systematic reviews and meta-analyses confirm that women with Lactobacillus-depleted microbiota exhibit a significantly higher risk of prematurity, reinforcing the protective role of vaginal eubiosis [73,74]. Mechanistically, this may involve ascending colonization, inflammatory activation, and cervical remodeling [75,142]. Vaginal dysbiosis is also associated with increased local and systemic inflammatory responses, including elevated pro-inflammatory cytokines and chemokines [75,142,143], which may disrupt cervical integrity and promote premature activation of parturition pathways. These inflammatory signals can extend to the maternal-fetal interface, influencing placental function, membrane stability, and fetal immune activation [143,144]. In this context, microbiome-driven inflammation represents a key pathway linking vaginal ecological imbalance to altered fetal development and early-life health outcomes [145].

Emerging evidence also links vaginal microbiome composition to infertility and early pregnancy loss. Lactobacillus-dominant communities are associated with improved embryo implantation and pregnancy success, while increased microbial diversity and dysbiosis correlate with reduced fertility and higher miscarriage risk [76,77] via modulation of endometrial receptivity [76,77]. The vaginal ecosystem is highly dynamic and influenced by hormonal and behavioral modulators, including estrogen levels, menstrual cycle phase, sexual activity, hygiene practices, and antibiotic exposure. Non-aggressive intimate hygiene preserves microbial balance, as harsh practices may disrupt Lactobacillus dominance and pH [18,75,78,79,80,81,82,83,84,85,86,87,88,89,90,91,92,93,94,95,96,97,98,99,100,101,102,103,104,105,106,107,108,109,110,111,112,113,114,115,116,117,118,119,120,121,122,123,124,125,126,127,128,129,130,131,132,133,134,135,136,137,138,139,140,141,142,143,144,145,146]. Estrogen-driven glycogen availability supports Lactobacillus proliferation, promoting microbial stability [147]. Within the integrated systems framework (Section 6), these modulators shape microbial structure and link macro-environmental exposures to mucosal immune homeostasis. Preventive strategies aim to preserve Lactobacillus dominance and limit dysbiosis [148]. Evidence remains evolving, but rational antibiotic use and microbiome-targeted interventions (including *L. crispatus* probiotics) have been proposed [18,79,80,81,82,83,84,85,86,87,88,89,90,91,92,93,94,95,96,97,98,99,100,101,102,103,104,105,106,107,108,109,110,111,112,113,114,115,116,117,118,119,120,121,122,123,124,125,126,127,128,129,130,131,132,133,134,135,136,137,138,139,140,141]. From a One Health perspective, these approaches may reduce inflammation-driven reproductive risks by stabilizing host–microbiome interactions [146].

### 4.4. Nutrition-Gut Microbiome Axis

Diet represents an important ecological determinant capable of modulating maternal gut microbial ecology and downstream metabolic and immune signaling pathways. Numerous scientific studies investigate the role of maternal diet on fetal programming and its interaction with the maternal gut microbiome. Kunasegaran T et al. [80] found that increased fiber intake in pregnant mice increases SCFA production, which, in turn, acts on specific receptors in the embryo, supporting fetal growth. Conversely, low fiber intake has been associated with reduced intestinal microbial biodiversity [81]. Westernized dietary patterns rich in ultra-processed foods, refined sugars, and high-fat content have been associated with reduced microbial diversity and pro-inflammatory microbial profiles [80]. Moreover, High-fat diets (HFDs) during pregnancy also appear to increase systemic inflammation in pregnant women, potentially affecting their intrauterine environment [83,84]. This factor should be taken into consideration when carbohydrate restriction in favor of lipids is indicated in women with gestational diabetes mellitus (GDM), as it may influence intestinal permeability and endotoxin-related signaling pathways, although evidence remains limited and not fully consistent [85].

Overall, maternal dietary patterns may influence microbial metabolite production and maternal-fetal signaling pathways involved in developmental programming [18,27,75,84,85,86,87,88,89,90,91,92,93,94,95,96,97,98,99,100,101,102,103,104,105,106,107,108,109,110,111,112,113,114,115,116,117,118,119,120,121,122,123,124,125,126,127,128,129,130,131,132,133,134,135,136,137,138,139,140,141,142,143,144,145,146,147,148,149]. Interventional approaches targeting the maternal gut microbiome are increasingly being explored. Prebiotics, including dietary fibers and resistant starches, can selectively promote the growth of beneficial microbial taxa and enhance the production of SCFA [149]. Microbiome-targeted nutritional strategies during pregnancy remain under investigation, and further studies are needed to clarify their long-term impact on maternal-fetal outcomes.

### 4.5. Gut Microbiome Metabolites as Mediators of Fetal Programming

Research in recent years has suggested a possible interaction between the maternal gut microbiota, the placental environment, and fetal development, with potential effects on pregnancy outcomes. Rather than direct microbial colonization, current evidence increasingly supports a model in which microbiota-derived metabolites and signals mediate this interaction. Some authors hypothesize that the fetus’s initial exposure to microorganisms and metabolites may occur prenatally. Microbial transfer could occur through various routes: hematogenously from the maternal oral microbiota, ascending from the vaginal tract, or through translocation from the maternal gut microbiota mediated by dendritic cells. These components could reach the amniotic fluid and, through their swallowing by the fetus, come into contact with the fetal intestine [87].

However, regardless of the presence of an intrauterine microbiota, metabolites produced by the maternal gut microbiota play a significant role in fetal development. These include B vitamins, folic acid, choline, betaine, polyphenols, and SCFAs derived from the fermentation of dietary fiber [18,75,88,89,90,91,92,93,94,95,96,97,98,99,100,101,102,103,104,105,106,107,108,109,110,111,112,113,114,115,116,117,118,119,120,121,122,123,124,125,126,127,128,129,130,131,132,133,134,135,136,137,138,139,140,141,142,143,144,145,146,147,148,149]. These molecules may contribute to the metabolic and nutritional programming of the fetus through epigenetic mechanisms and neurochemical signaling [27]. Some metabolites can cross the placenta or act at the maternal-fetal interface, potentially contributing to central nervous system development and immune maturation. The maternal gut microbiota is also involved in the production and modulation of neuroactive compounds such as serotonin (5-HT) and gamma-aminobutyric acid (GABA) [91]. At the same time, it is important to recognize that these pathways are closely interconnected with host neuroendocrine mechanisms, including maternal stress responses and cortisol signaling, which may independently or synergistically influence fetal development. Evidence from microbiota-deficient (GF) mouse models shows alterations in hippocampal gene expression, affecting neurotransmission, neuroplasticity, and metabolism [150].

However, these findings also highlight the difficulty of disentangling microbiome-specific effects from broader host regulatory systems, including tryptophan metabolism and stress-related pathways within the gut–brain axis.

Overall, current evidence supports a role for maternal microbiota-derived metabolites as potential modulators of fetal developmental processes, but causal relationships remain to be fully elucidated. Greater clarity is needed to distinguish direct microbiome-mediated effects from those driven by host endocrine and metabolic responses, particularly in the context of maternal stress and its downstream signaling pathways.

### 4.6. Mode of Delivery as an Ecological Transition Event

Birth is a true ecological transition marking the newborn’s passage from the intrauterine environment to an external microbial ecosystem. During this critical window, the mode of delivery shapes early microbial exposures and contributes to the initial configuration of the neonatal ecosystem, with effects extending beyond the perinatal period [94]. As outlined in Section 3.1, delivery mode is associated with distinct patterns of microbial transmission and early colonization, reflected in differences in key taxa such as *Bifidobacterium* and *Bacteroides* and in overall community structure [95,96]. From a One Health systems perspective, mode of delivery represents an early-life ecological determinant that integrates maternal physiology, clinical decision-making, and environmental microbial exposures. Within a systems perspective, mode of delivery acts as an initial ecological imprint that interacts with postnatal determinants, potentially amplifying or mitigating early microbial trajectories [26,27]. Early microbial colonization occurs within a critical window of immune development, contributing to the maturation of host responses and the establishment of immune tolerance. These early microbial configurations may influence fetal-derived immune programming trajectories transitioning into neonatal lifeshaping of developmental trajectories [88].

Over recent decades, the cesarean section rate has increased worldwide, reaching 21.1% of births, with projections reaching and projected to rise to 28.5% by 2030 [97]. Infants born by cesarean section exhibit a distinct microbiota composition and, in some cases, an increased risk of immune-mediated and metabolic conditions. However, these associations should be interpreted with caution, considering the role of confounding factors and the predominantly observational nature of the available evidence [98,99]. Within this framework, mode of delivery represents a clinically modifiable determinant embedded in a broader ecological and healthcare system, where medical practices and decision-making processes shape early microbial exposures. Emerging strategies aimed at mitigating cesarean-associated dysbiosis, including optimized breastfeeding and microbial restoration approaches, are under investigation but not yet standardized [151].

### 4.7. Physical Activity and Microbial Ecology

Physical activity represents a modifiable ecological determinant capable of influencing maternal microbial ecology and associated metabolic and inflammatory pathways during pregnancy [132]. Physical activity is associated with increased gut microbial alpha diversity, with the strongest effects observed in aerobic and combined training protocols [100].

Recent meta-analyses confirm significant enrichment of SCFA-producing taxa such as *Faecalibacterium*, *Roseburia*, and *Veillonella*, the latter converting lactate to propionate during endurance exercise [100,101]. Exercise-driven microbial changes correlate with reduced low-grade inflammation and improved metabolic homeostasis. SCFAs, especially butyrate, contribute to intestinal barrier function and immune regulation [102]. These exercise-associated microbial adaptations may contribute to maternal metabolic regulation and inflammatory homeostasis during pregnancy [101,102,103,104].

Importantly, the impact of physical activity on the microbiome appears to be dose- and context-dependent, influenced by intensity, duration, and baseline host metabolic status. Overall, physical activity may represent a relevant non-pharmacological strategy for supporting maternal microbial and metabolic homeostasis during pregnancy.

### 4.8. Neuroendocrine Stress Axis and Microbiota

The activation of the hypothalamic–pituitary–adrenal (HPA) axis constitutes a central component of maternal adaptation to stress during pregnancy. Glucocorticoids, particularly cortisol, can cross the placental barrier, thereby modulating the development of the fetal neuroendocrine system [110]. The secretion of these molecules is induced by stressful stimuli that occur during the gestational phase [1,2,16,17,18,19,20,21,22,23,24,25,26,27,28,29,30,31,32,33,34,35,36,37,38,39,40,41,42,43,44,45,46,47,48,49,50,51,52,53,54,55,56,57,58,59,60,61,62,63,64,65,66,67,68,69,70,71,72,73,74,75,76,77,78,79,80,81,82,83,84,85,86,87,88,89,90,91,92,93,94,95,96,97,98,99,100,101,102,103,104,105,106,107,108,109,110,111,112,113,114,115,116,117,118,119,120,121,122,123,124,125,126,127,128,129,130,131,132,133,134,135,136,137,138,139,140,141,142,143,144,145,146,147,148,149,150,151,152]. Cortisol also plays a modulatory role in intestinal homeostasis by acting on intestinal barrier permeability and immune signaling pathways, thereby influencing nutrient availability and microbial niche composition [1,2,16,17,18,19,20,21,22,23,24,25,26,27,28,29,30,31,32,33,34,35,36,37,38,39,40,41,42,43,44,45,46,47,48,49,50,51,52,53,54,55,56,57,58,59,60,61,62,63,64,65,66,67,68,69,70,71,72,73,74,75,76,77,78,79,80,81,82,83,84,85,86,87,88,89,90,91,92,93,94,95,96,97,98,99,100,101,102,103,104,105,106,107,108,109,110,111,112,113,114,115,116,117,118,119,120,121,122,123,124,125,126,127,128,129,130,131,132,133,134,135,136,137,138,139,140,141,142,143,144,145,146,147,148,149,150,151,152]. Recent scientific evidence suggests a correlation between persistent hyperactivation of the fetal HPA axis and the production of immune and metabolic mediators that lead to alterations in placental nutrient transfer, resulting in potentially significant neurodevelopmental changes [111,112]. Microbial metabolites, including SCFAs and tryptophan-derived compounds, may act as intermediaries between stress signaling and host physiology [153].

Overall, maternal stress and microbiome dynamics should be interpreted as interconnected components of a broader neuroendocrine-immune regulatory network influencing fetal programming [154].

### 4.9. Antibiotic Exposure as a Major Iatrogenic Perturbation of the Maternal-Infant Microbial Axis

Antibiotic exposure represents a cross-cutting determinant capable of disrupting multiple microbial ecosystems simultaneously, with downstream effects across several pathways described in this section. Antibiotic exposure during pregnancy, during childbirth, and in the postpartum period represents the main iatrogenic determinant of alteration of the maternal-infant microbial axis, with relevant implications in the field of fetal programming [117]. Clinical indications for antibiotics include maternal infections, intrapartum prophylaxis, and obstetric complications; however, even short exposures rapidly alter maternal intestinal, vaginal, cutaneous, and mammary microbiota [118,119]. In addition, several commonly used antibiotics administered during pregnancy or intrapartum are known to cross the placenta and reach the fetal compartment, resulting in direct fetal exposure [155]. These modifications result in reduced microbial diversity, selection of resistant opportunistic taxa, and disruptions of microbial functional and metabolic activity [119]. Antibiotics, therefore, act as ecological selective pressures that reshape microbial community trajectories rather than merely reducing bacterial load. These alterations of maternal microbial ecosystems, together with direct fetal exposure to antibiotics via transplacental transfer, may influence early-life microbial assembly by disrupting vertical transmission processes during birth and breastfeeding. This, in turn, has the potential to alter postnatal gut colonization and modulate early immune and metabolic responses in the newborn [120]. Effects extend beyond taxonomic composition to key microbial functions, including short-chain fatty acid production, immune modulation, and barrier integrity [121].

In this context, the need for a conscious clinical practice suitable for the care of the microbiota emerges, which includes a prudent and targeted use of antibiotics, integrated with microbial modulation strategies such as prebiotics, probiotics, and postbiotics. The efficacy of these interventions depends on strain, timing, and biological compatibility with antibiotics [122]. In particular, commonly used β-lactam antibiotics in pregnancy (amoxicillin, amoxicillin–clavulanate, ampicillin) can reduce the viability of certain probiotics [122]. Although many *Bifidobacterium* species are generally sensitive to β-lactams, some strain-specific differences in susceptibility have been reported, including *Bifidobacterium breve* PRL2020, which has been characterized in vitro as showing reduced susceptibility to amoxicillin and amoxicillin-clavulanate, with minimal inhibitory concentration values suggesting tolerance in laboratory susceptibility testing [123]. In contrast, spore-forming probiotics such as *Bacillus clausii* and *Clostridium butyricum* are relatively resilient in the presence of antibiotics [124,125]. Furthermore, commonly widely used drugs such as proton pump inhibitors show non-negligible effects on the composition of the maternal microbiota, amplifying the risk of dysbiosis [126].

### 4.10. Postnatal Continuity of Microbial Transmission

Beyond prenatal microbial signaling, the postnatal period represents a modifiable ecological interface where maternal-infant microbial continuity is actively shaped. The postnatal period is a critical window where maternal microbes shape infant health, with modifiable factors supporting a dynamic mother-infant microbial axis. Skin-to-skin contact is crucial for newborn health, improving physiological stability, thermoregulation, and behavioral regulation. Skin-to-skin contact is a key early-life exposure supporting neonatal adaptation and facilitating maternal microbial transfer. It enhances autonomic maturation, influencing immune function and gut microbiota development [128]. Physical contact also promotes maternal cutaneous and oral microbial transfer, supporting commensal colonization and limiting opportunistic pathogens [18]. Even without breastfeeding, close contact remains an effective strategy to support neonatal microbial and immunological development [129]. Breastfeeding is the gold standard for infant nutrition as a dynamic vehicle for a wide range of bioactive components [130] and a cornerstone of postnatal microbial transmission. Its composition evolves from colostrum to mature milk, adapting to infant needs. Milk microbiota arises from enteromammary transfer, retrograde flow from the infant’s mouth, and possible oromammary routes and is shaped by maternal and environmental factors. It consists of a highly diverse microbial community [131] and reflects maternal health, antibiotic use, BMI, and feeding mode, with direct breastfeeding promoting beneficial oral-derived microbes and greater microbial diversity, influencing health outcomes, including reduced infections and allergies. A key driver of this selective microbial enrichment is represented by human milk oligosaccharides (HMOs), which act as prebiotics and selectively promote the growth of beneficial taxa such as Bifidobacteria [132]. In addition to HMOs, lactose also contributes to shaping the early gut microbiome by serving as a fermentable substrate with prebiotic effects, thereby supporting the establishment of a favorable microbial ecosystem [133]. Collectively, these components act synergistically in modulating early microbial colonization and immune development. Interventions targeting these modifiable factors can optimize microbial colonization, support immunity, and reduce chronic disease risk within a One Health framework.

## 5. Macro-Environmental Determinants and Epigenetic Modifications in Maternal Microbial Ecology: A One Health Perspective

Within a One Health-informed systems perspective, macro-environmental exposures should be interpreted as interconnected ecological determinants converging on shared maternal microbial, immune, metabolic, and placental pathways. The maternal microbiome represents a key interface through which environmental and lifestyle-related exposures may influence maternal-fetal signaling and early developmental trajectories [156].

These determinants include urbanization, pollutants, dietary habits, physical activity patterns, psychosocial stress, medical exposures, and environmental microbial contact, all of which may interactively shape maternal microbial ecology and downstream maternal-fetal signaling pathways [156].

A summary of these determinants, their gut microbiota effects, mechanisms, and maternal-fetal outcomes is presented in Table 2.

Urbanization and related lifestyle changes are major drivers of microbiome disruption. Reduced exposure to natural environments, increased hygiene, sedentary behaviors, and circadian disruption are associated with decreased microbial diversity and resilience, promoting a Westernized microbiome characterized by lower diversity, a higher *Firmicutes*/*Bacteroidetes* ratio, and enrichment of pro-inflammatory taxa [153]. Collectively, dietary patterns, sedentary behaviors, psychosocial stress, medical interventions, and reduced environmental biodiversity may contribute to cumulative ecological pressures capable of altering maternal microbial stability and immune-metabolic homeostasis.

In maternal populations, these exposures have been associated with reduced microbial diversity and taxonomic shifts, potentially affecting both gut and breast milk microbiota composition [154,157]. These alterations may influence the transfer of microbial and immune signals to the infant, although causal links with long-term outcomes remain to be fully established.

Chemical pollutants also interact with host and microbial biology. Pollutants such as air contaminants (PM2.5, NO_2_), heavy metals, and persistent organic pollutants can alter microbial composition and host responses, including gut permeability and immune activation [158,159]. In addition, air pollution has been linked to gut dysbiosis through the lung-gut axis [160,161]. Environmental pollutants have additionally been associated with epigenetic modifications in maternal tissues, suggesting potential interactions between environmental stressors, microbial ecology, and fetal developmental programming, although causal mechanisms remain incompletely defined.

Emerging contaminants such as microplastics have been detected in human tissues, including the placenta, but their specific effects on the maternal microbiome and fetal development are still under investigation and remain largely speculative.

People spend much time indoors, making the indoor microbiome a key source of exposure. Household microbial diversity, shaped by building materials, ventilation, pets, and human occupants, can support maternal microbiota and immune tolerance, while overly sanitized environments may promote dysbiosis [162,163]. Lifestyle-related determinants may also influence substrate availability for beneficial microbes and modulate inflammatory signaling pathways relevant to maternal-fetal homeostasis [169,170]. People spend much time indoors, making the indoor microbiome a key source of exposure. Household microbial diversity, shaped by building materials, ventilation, pets, and human occupants, can support maternal microbiota and immune tolerance, while overly sanitized environments may promote dysbiosis [162,163].

Collectively, these environmental and lifestyle-related exposures should be interpreted as upstream ecological modulators acting through interconnected microbial, immune, metabolic, and epigenetic pathways [164]. Although several mechanistic pathways remain incompletely characterized, current evidence supports the integration of environmental and lifestyle determinants into microbiome-mediated models of maternal-fetal programming and early-life health.

## 6. An Integrated Systems Model of Microbiome-Mediated Maternal-Fetal Programming

**Figure 1:**
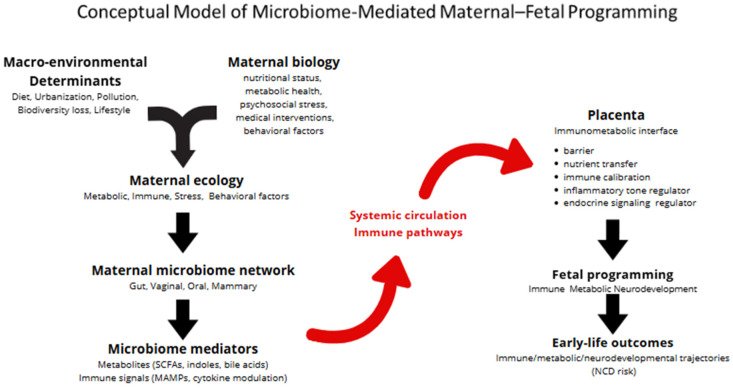
Pregnancy as an eco-biological system linking environment, maternal microbiome, and fetal programming.

Pregnancy may therefore be viewed as an integrated eco-biological system in which macro-environmental conditions, maternal ecology, and microbiome-mediated signaling pathways collectively contribute to shaping the intrauterine environment [16] (Figure 1).

The maternal microbiome acts as a dynamic network that integrates environmental and host-derived signals and translates them into circulating metabolites and immune mediators that may influence fetal development [1,2,13,14,15,16,17,18,19,20]. The placenta functions as an immunometabolic interface integrating maternal metabolic and immune cues into coordinated fetal responses affecting immune, metabolic, and neurodevelopmental trajectories [3,15,24,38].

Collectively, this systems model integrates environmental exposures, maternal microbiome dynamics, placental function, and fetal developmental programming within a unified maternal-fetal ecological framework.

The maternal microbiome acts as a signaling hub, producing metabolites and immune-modulatory factors that reach the placenta via systemic circulation or indirectly modulate maternal pathways. Together, these processes contribute to fetal immune, metabolic, and neurodevelopmental programming across early-life windows, ultimately shaping health trajectories and the risk of non-communicable diseases.

## 7. Limitations of Current Evidence

Despite increasing interest in microbiome-mediated maternal-fetal programming, current evidence is constrained by several important limitations.

Limited causal inference remains a central issue. Most human studies are observational, identifying associations between maternal microbiota and offspring outcomes without establishing causality. Although animal models provide mechanistic insights, interspecies differences in physiology, immune development, and microbiome composition limit direct translation to humans.

Moreover, confounding variables further complicate interpretation. Maternal diet, metabolic status, antibiotic exposure, socioeconomic factors, delivery mode, and breastfeeding practices all influence both microbiota and health outcomes. In addition, host genetic variation represents a significant and often underappreciated confounding factor, as genetic background can influence both microbiome composition and host susceptibility to disease. These interconnected variables are difficult to control fully, raising the risk of residual confounding and potential overestimation of microbiome-specific effects [171]. Therefore, methodological heterogeneity across studies limits comparability. Variations in sampling sites, timing, sequencing techniques, and analytical pipelines contribute to inconsistent findings. In addition, many studies focus on taxonomic composition rather than functional outputs, such as metabolomic or transcriptomic profiles, despite evidence that microbial metabolites may play a more direct role in host physiology.

The lack of longitudinal studies represents another key limitation. Most data derive from cross-sectional or limited timepoint analyses, which fail to capture the dynamic nature of microbiome changes across pregnancy and early life. Long-term follow-up of offspring is also limited, restricting the ability to link early microbial exposures with later health outcomes.

Finally, significant translational gaps between animal and human research persist. Experimental models offer controlled conditions that do not reflect the complexity of human environments. As a result, microbiome-targeted interventions that show promise in preclinical studies often yield inconsistent results in clinical settings and are not yet part of routine obstetric care.

Collectively, these limitations highlight the need for standardized methodologies, longitudinal designs, and multi-omics approaches, particularly those focusing on microbial functional outputs and metabolite-mediated signaling, to strengthen causal inference and support clinical translation.

## 8. Future Directions

Advancing the understanding of microbiome-mediated maternal-fetal programming requires integration of mechanistic, translational, and clinical research. A critical priority for advancing the field is the development and adoption of alternative experimental models that better reflect human-specific maternal-fetal interactions. While traditional animal models have provided valuable mechanistic insights, their translational relevance is limited by interspecies differences in microbiome composition, placentation, immune development, and metabolic regulation. Emerging approaches include human-relevant in vitro systems such as placental organoids [169], which allow controlled investigation of maternal-fetal interactions. Complementary insights into fetal immune development and regulation are provided by studies on human fetal immune systems [170]. In parallel, longitudinal human cohort studies integrating multi-omics and strain-level microbial tracking represent a complementary strategy to capture real-world complexity and maternal-infant microbial transmission dynamics. Together, these approaches may provide more accurate and clinically translatable insights into microbiome-mediated fetal programming. Although evidence supports a role for maternal microbial ecosystems in early-life development, key areas remain underexplored. Microbiome-targeted interventions during pregnancy, including dietary modulation, prebiotic and probiotic supplementation, and lifestyle strategies, have shown potential to influence maternal microbial composition and metabolite profiles, though randomized trial evidence remains limited and heterogeneous [1,2,17,18,19,20,21,22,23,24,25,26,27,28]. Such interventions could leverage vertical transmission of beneficial microbes, including bifidobacterial communities, to support neonatal colonization and immune development [172] and may mitigate risks associated with Western dietary patterns [51].

Precision medicine approaches are critical given inter-individual variability in microbiota, host genetics, and environmental exposures. Integration of multi-omics datasets, metagenomics, metabolomics, and epigenomics can help identify individualized microbial and metabolic profiles linked to pregnancy outcomes [5]. Translating these approaches clinically will require standardized methodologies, robust cohorts, and ethical oversight.

Microbiome-based biomarkers hold promise for early risk stratification. Specific microbial and metabolite signatures have been associated with adverse pregnancy outcomes and infant health trajectories, including allergy, metabolic disorders, and neurodevelopmental risk [25,26,27,28,29,30,31,32,33,34,35,36,37,38,39,40,41,42,43,44,45,46,47,48,49,50]. Prospective longitudinal studies are needed to confirm predictive validity and establish clinical utility.

From a public health perspective, early-life microbial exposures influence immune and metabolic programming relevant to asthma, obesity, and neurodevelopmental conditions [18,26,27,28,29,30,31,32,33,34,35,36,37,38,39,40,41,42,43,44,45,46,47,48,49,50,51,52,53,54,55,56,57,58,59,60,61,62,63,64,65,66,67,68,69,70,71,72,73,74,75,76,77,78,79,80,81,82,83,84,85,86,87,88,89,90,91,92,93,94,95,96,97,98,99,100,101,102,103,104,105,106,107,108,109,110,111,112,113,114,115,116,117,118,119,120,121,122,123,124,125,126,127,128,129,130,131,132,133,134,135,136,137,138,139,140,141,142,143,144,145,146,147,148,149,150,151,152,153,154,155,156,157,158,159,160,161,162,163,164,165,166,167,168,169,170,171,172]. Interventions targeting maternal and postnatal environments could reduce lifelong disease burden, although causal pathways require further clarification. Integrating microbiome research within a One Health framework emphasizes that individual-level interventions should be complemented by population-level strategies addressing environmental determinants of maternal and infant health [4].

Collectively, these priorities underscore the need for interdisciplinary, longitudinal, and systems-based research to bridge knowledge gaps and enable safe, effective, evidence-informed microbiome strategies in maternal and child health. Future advances will likely depend on integrating environmental, microbial, immunological, metabolic, and host genetic data into unified models of maternal-fetal health and developmental programming.

## 9. Conclusions

Pregnancy emerges as a transient yet highly integrated biological system shaped by maternal physiology, environmental factors, and the microbiome, driving fetal development and early-life health trajectories. The maternal microbiome, spanning gut, vaginal, oral, and mammary niches, functions as a regulatory hub influencing placental function and developmental programming through metabolic, immunological, and neuroendocrine pathways. These processes operate across developmental windows from preconception through gestation to early postnatal life.

Despite progress in characterizing microbial transmission routes, metabolite-mediated signaling, and placental integration of maternal cues, translation into clinical practice remains limited. Maternal microbial composition and activity act as modulators rather than deterministic drivers within a context-dependent framework, including diet and metabolite profiles linked to neurodevelopmental outcomes. Collectively, current evidence supports the interpretation of pregnancy as a modifiable ecological system in which maternal diet, lifestyle, microbial exposures, medical practices, and environmental conditions may influence developmental programming and early-life health trajectories within a One Health-informed framework.

Future research should prioritize longitudinal and multi-omics studies capable of integrating microbial, metabolic, immune, environmental, and placental data across developmental windows in order to clarify causal mechanisms and identify clinically relevant biomarkers. From a translational perspective, microbiome-informed prenatal strategies, including nutritional optimization, prudent antibiotic stewardship, personalized lifestyle interventions, and reduction in environmental risk exposures, may represent promising avenues for improving maternal and offspring health. At the population level, preventive strategies addressing urbanization-related lifestyle changes, dietary quality, pollution exposure, and environmental biodiversity may contribute to supporting intergenerational health trajectories.

Collectively, these perspectives reinforce the concept of microbiome-mediated maternal-fetal programming as a dynamic ecological process linking environmental conditions, maternal biology, and early-life developmental trajectories. Ultimately, integrating microbiome science into maternal-fetal medicine may contribute to the development of more preventive, individualized, and ecologically informed models of early-life healthcare.

## Figures and Tables

**Table 1:** Overview of the Ten Ecological Determinants of Maternal-Fetal Microbiome-Mediated Programming. The table summarizes each determinant, its impact on maternal microbiota composition, key microbial metabolites and mechanisms, effects on fetal and neonatal development, and potential prevention or intervention strategies. Key references supporting each determinant are also provided to ensure scientific rigor.

Ecological Determinant	Maternal Microbiota Effect	Key Metabolites Mechanisms	Fetal/Neonatal Impact	Prevention/Intervention	Key References
1. Maternal-fetal microbial signaling during pregnancy.	Dynamic remodeling of maternal microbial ecosystems and host-microbe interactions.	Microbial metabolites, immune factors, endocrine signaling, epigenetic modulation	Fetal programming, developmental plasticity, immune priming	Maternal diet, microbiome-friendly lifestyle	[22,23,24,25,26,27,28,29,30,31,32,33,34,35,36,37,38,39,40,41,42,43,44,45,46,47,48,49,50,51,52,53,54,55,56,57,58,59,60,61,62,63]
2. Oral microbiome-systemic inflammatory axis	Dysbiosis associated with systemic inflammation	LPS, pro-inflammatory cytokines, microbial translocation	Preterm birth, low birth weight, immune modulation	Oral hygiene, probiotics *(S. salivarius*, *L. reuteri)*	[64,65,66,67,68,69,70,71]
3. Vaginal microbiome	Lactobacillus-dominance promotes stability, dysbiosis increases inflammation	Lactic acid, low pH, antimicrobial peptides, immune modulation	Reduced preterm risk, improved fertility, vertical microbial transmission	Probiotics *(Lactobacillus. crispatus*), gentle hygiene practices	[72,73,74,75,76,77,78,79]
4. Nutrition-gut microbiome axis	Diet-driven modulation of microbial diversity and function	SCFAs, B vitamins, polyphenols, neurotransmitters	Neuroimmune development, metabolic programming	Balanced diet, fermented foods, resistant starches	[22,80,81,82,83,84,85,86]
5. Gut microbiome metabolites and signaling pathways.	Functional metabolic output of gut microbiota	SCFAs, serotonin, GABA, folate, choline, tryptophan derivates	CNS development, immune maturation, metabolic setpoint	Diet, stress management, limiting unnecessary antibiotics	[87,88,89,90,91,92,93]
6. Mode of delivery and early microbial seeding	Initial neonatal colonization patterns differ by delivery mode	*Bifidobacterium*, *Bacteroides*, microbial succession	Immune and metabolic programming, allergy risk	Vaginal delivery, microbial restoration, optimized breastfeeding	[22,94,95,96,97,98,99]
7. Physical activity microbiome interactions	Increased gut microbial diversity and metabolic capacity	SCFA-producing taxa, myokine-microbiome interactions	Reduced systemic inflammation, improved metabolic environment	Personalized exercise programs	[100,101,102,103,104,105,106,107,108,109]
8. Stress-microbiome neuroendocrine interactions	Stress-induced dysbiosis via HPA axis activation	Cortisol, catecholamines, altered microbial metabolites	Altered neurodevelopment, stress susceptibility	Stress management, psychosocial support	[16,110,111,112,113]
9. Antibiotics, medications and microbiota-modulating esposures	Reduced diversity, compositional shifts or selective enrichment depending on exposure	Altered metabolites, barrier integrity, immune signaling	Altered neonatal colonization, immune and metabolic programming	Prudent prescribing, evidence-based supplementation, personalized counseling	[114,115,116,117,118,119,120,121,122,123,124,125,126]
10. Mammary microbiome and postnatal microbial continuity	Breast microbial communities and maternal-infant transfer pathways	HMOs, bifidobacteria, commensal milk microbes, immune factors	Neonatal colonization, immune priming, metabolic maturation	Breastfeeding support, maternal nutrition, prudent antimicrobial use	[18,20,36,127,128,129,130,131,132,133]

**Table 2:** Summary of macro-environmental determinants influencing maternal microbial ecology, including their effects on gut microbiota composition, underlying mechanisms, and potential maternal-fetal outcomes. The table highlights how environmental exposures may act as upstream ecological modulators within microbiome-mediated maternal–fetal programming.

Macro-Environmental Determinant	Gut Microbiota Effects	Key Mechanisms	Maternal & Fetal Health Outcomes	References
Urbanization & Lifestyle Changes	Reduced diversity, increased Firmicutes/Bacteroidetes ratio, higher pro-inflammatory taxa, altered breast milk microbiota	Limited environmental microbial exposure, Westernized diet, cesarean delivery, antibiotic use, chronic stress, circadian disruption leading to immune and metabolic dysregulation.	Increased risk of obesity, metabolic disorders, allergies, infections, impaired immune signaling	[153,154,157,158,159,160,161,162,163,164,165]
Industrialized Diets/Ultra-Processed Foods (UPFs)	Reduced microbial diversity, decreased SCFA production, increased pathobionts	Low fiber intake, high fat and sugar content, and exposure to food additives and xenobiotics, resulting in reduced SCFA production, increased inflammation, and altered gut permeability	Gestational diabetes, excessive maternal weight gain, inflammation, poor neonatal metabolic outcomes, infant adiposity	[154,158,159,160,161,162,163,164,165,166,167]
Environmental Pollutants (PM2.5, NO_2_, heavy metals, POPs)	Gut dysbiosis, altered microbial metabolism	Exposure to inhaled or ingested pollutants contributing to oxidative stress, increased gut permeability, immune activation, and microbial biotransformation of toxic compounds	Maternal cardiovascular/respiratory effects, systemic inflammation, placental dysfunction, fetal programming, long-term metabolic and immune risks	[158,159,160]
Microplastics/Nanoplastics	Altered microbial composition, bioaccumulation in tissues	Ingestion of particles associated with oxidative stress, inflammatory responses, disruption of gut barrier integrity, and potential epigenetic modulation	Potential contribution to metabolic dysregulation, chronic inflammation, and long-term disease susceptibility in offspring (emerging evidence)	[168]
Household Microbial Environment	Modulation of microbial diversity, and composition, promotion of beneficial taxa	Environmental exposures (pets, cohabitation, ventilation, building materials, hygiene practices) influencing immune education and microbial exchange	Improved immune tolerance, reduced allergy risk, enhanced neonatal microbial colonization, and improved immune development	[162]
Epigenetic/Transgenerational Effects	Persistent microbiome alterations across generations	DNA methylation, histone modifications, microRNA regulation influenced by environmental and microbial signals	Increased susceptibility to metabolic, immune, and neurodevelopmental disorders across generations	[156]

## Data Availability

No new data were created or analyzed in this study. Data sharing is not applicable to this article.
